# "The Hospital" Nursing Mirror

**Published:** 1896-05-09

**Authors:** 


"The Hospital" Nursing Mirror.

Being the Extra Nursing Supplement of "The Hospital" Newspaper.

[Contributions for this Supplement should be addressed to the Editor, The Hospital, 428, Strand, London, W.C., and should have the word "Nursing" plainly written in left-hand top corner of the envelope.]

### News from the Nursing World.

### A ROYAL HOSPITAL.

Queen Wilhelmina of the Netherlands takes much interest in the fine hospital called by her name at The Hague. The Queen laid the foundation-stone of the Wilhelmina Hospital herself a few years since, and when she is at The Hague frequently pays a personal visit to its bright and cheerful wards, speaking kindly words to the patients, to whom her presence is a real pleasure. There is a great deal of enthusiasm among the Dutch people for their youthful Sovereign, whom they hold in affectionate admiration.

### PROPOSED FEDERATION OF NURSING ASSOCIATIONS.

A proposal is on foot to form a federation in Nottinghamshire of all the nursing associations in the county, with the idea of representing "all systems of district nursing, the functions of such federation to be to consider questions of policy and general relations of district nursing to public bodies, the organisation and conduct of the business of each federated association to be left in the hands of each association." We note, however, that Mr. Dunstan, Director of Technical Instruction for the County Council, the propounder of the scheme, proposes that all systems of district nursing, "whether short-trained or long-trained," shall be represented, and this means the formal recognition of those women who avail themselves of the County Council's scholarships for a period of training of six months only. If these women, who can in no sense be considered "nurses" in any proper meaning of the term, are to be turned loose upon the public under that designation, a grave danger seems unavoidable. A great confusion of standards is certain to result, ultimately leading to the adoption of a generally lowered level all round.

### A REFUGE FOR "INCURABLES."

St. Catherine's Home for Cancer and Incurables, Bradford, is one of those institutions, of which there are far too few, intended to afford a last refuge for cases unsuitable for ordinary hospitals. It is for women only, and "no recommendations are necessary beyond those of sickness and distress, neither character nor creed, nor the absence of either or both, being a bar to admission." The work of mercy so quietly and unostentatiously carried on in this little home deserves every support, and needs just now help to enable an at present empty ward of four beds, recently furnished as a gift from Mr. F. Illingworth, to be put into permanent use. It seems, indeed, a pity that this generous gift should be to no purpose for want of the extra £180 required to defray the cost of occupation. Though only established three years ago, the value and need of St. Catherine's is recognised on all sides, and next year will participate in the Joint Hospital Fund. A donation has also been given from the fever hospital trustees. The Home owes much to Miss Hadley Scott's good management and kindly care as matron.

### A WISE PROVISION FOR THE FUTURE.

The committee of the Birmingham and Midland Homœopathic Hospital and Dispensary have decided that it is desirable for the hospital to become affiliated to the Royal National Pension Fund for Nurses, and have therefore arranged "to set apart out of the moneys obtained as nurses' and nursing fees sufficient to pay the premium for sick pay of 15s. per week, and half the premium for a pension at the age of 55 of £22 10s. per annum for nurses who may have been in the hospital for at least twelve months, and who are eligible to become members of the fund." The annual report, from which we quote, adds, "The second half of the pension is payable by the nurses themselves, and by this means a method is secured of helping those to whom sick folk owe so much to help themselves, and the effect of which may prove far-reaching and beneficial." This step proves that the authorities of the Birmingham Homœopathic Hospital have the best interests of their nursing staff at heart, and we congratulate them on their wise and provident action.

### PATHOLOGICAL SOCIETY AND A LADY MEMBER.

A qualified medical woman has applied to the Pathological Society of London to be admitted a member of the society, and the matter is to be decided at the annual general meeting on May 16th. The bye-law of the society on the subject of members' qualifications states that "Physicians, surgeons, general practitioners, and persons distinguished in other departments of science connected with medicine shall be eligible for nomination as members." No disqualification can be held to be implied in these words, and the decision of the society will be awaited with interest.

### DIFFICULTIES AT BATH.

In the light of the attention which has lately been called to the condition of nursing in workhouse infirmaries, and the position of nurses under the present Poor Law system, the reports in the local press of the last meeting of the Bath Board of Guardians contain some instructive reading. It would seem that resignations have been frequent lately among the nurses, and a special committee was appointed to inquire into the causes of dissatisfaction, the report being presented at the meeting in question. One of the Guardians remarked that the information vouchsafed was "delightfully vague, and it was clear that though something was wrong the committee did not mean to let them know what it was." The position of the committee, and at the same time of the nurses, seems to be briefly summed up in the reply made to a question as to why nurses had left the workhouse. "Mr. King replied that the committee resolved not to go into that, because the master and matron being irremovable it would be undesirable to take evidence likely to discredit their authority!"

### A LEGACY FROM MRS. GAMP.

THERE are still a few hospitals, of which better things might be expected, where the objectionable custom prevails of allowing the nurses to make their own afternoon tea *in* the wards, so that a chance visitor may be struck by the incongruous spectacle of nurses seated at a daintily-spread tea-table a few feet from the bed of a dying patient. Of course this system is a relic from the days when nurses were of the charwoman class, and had allowances of rations to cook and eat how and where they chose; but from every possible point of view, hygienically as well as sentimentally, there can be no excuse for a continuance of this arrangement. It is disgusting for the nurses, and unwholesome too. Afternoon tea should be served in the nurses' dining-room in the same way as any other meal, between certain hours, to allow of the nurses going in relays as may be found convenient.

### WALTHAMSTOW.

THE Children's and General Hospital, Walthamstow, E., which at present contains twenty beds, is about to be enlarged by the addition of another twenty, and the accommodation for the nursing staff is also to be increased and improved upon. In the recently issued annual report the Council pay a special tribute of acknowledgment to Miss Hunter, who was appointed matron in 1894, for "the way in which she has organised the working of the hospital, and overcome the many difficulties and worries inseparable from the task of starting a practically new institution."

### NURSING IN NEW ZEALAND.

MISS SQUIRE, the recently-appointed matron of the Auckland Hospital, New Zealand, has been having six months of heavy work. Enteric fever has been unusually prevalent lately, and three of the nursing staff have fallen victims to it, their nursing adding, of course, much to the matron's anxieties, especially as it has been impossible to supply their places in the wards. The Auckland Hospital has gone through stormy times in past years, and there have been many tales of difficulties and dissensions amongst the medical and nursing staffs, the internal atmosphere of the hospital appearing to be of a volcanic nature like the island. It must be hoped that peaceful times are now ahead, and that the alterations which are being carried out will result in an improved condition of things all round.

### NURSING AT BRISTOL.

THE Bristol District Nursing Society was able to present a very satisfactory report at the annual meeting held last month. The great event of the past year has been the purchase of a permanent home for the nurses of the society, a step which has been possible through a legacy of £1,000, free of duty, bequeathed by the late Mr. Samuel Jones, for many years a liberal supporter and warm friend. The greater part of this money has been invested in the purchase of No. 6, Berkeley Square, a house which by association seems to have a special fitness for its new use. In it lived and died Miss Laura Edwards, one of the first organisers of district nursing in Bristol, and who was to the end of her life keenly interested in the work. This new and increased accommodation will enable the staff of nurses to be added to as occasion arises. There are now, in addition to the district nurses, whose work lies solely among the poor, two private nurses, whose services are proving very satisfactory, and there is also a "daily nurse," who visits cases where a resident nurse is not required, and attends operations. It is interesting to note, in connection with some letters on this subject which have recently appeared in these columns, that the committee report that "this plan of a daily nurse has met with much appreciation both by medical men and patients." The committee specially put on record their deep sense of the good work done by Miss Lloyd, the lady superintendent. The financial statement showed that the total receipts for the past year amounted to £1,715 4s. 11d., and the expenditure, including a deficit from 1894 of £54 10s. 6d., was £1,705 0s. 1d., leaving a balance in hand of £10 4s. 10d.

### SHORT ITEMS.

THE annual meeting of the Factory Girls' Country Holiday Fund was held at the Mansion House on April 28th. Its object is a most excellent one, the fortnight's summer holiday in the country bringing fresh life and strength to many weary London workers, and every small contribution will be gratefully received by the hon. secretary, Miss Canney, St. Peter's Rectory, Saffron Hill, E.C. — The new Passmore Edwards Cottage Hospital at Liskeard, just opened, is for the future to be also the headquarters of the District Nursing Association.—Miss May Abraham, senior lady inspector of factories, has been appointed by the Home Secretary, Sir Mathew White Ridley, to the rank of superintending inspector.—A bazaar and sale of work in aid of the District Nurses' Home at Plaistow, of which Sister Katherine is the well-known and energetic head, was held the last week in April at the Public Hall, Canning Town.—On Tuesday, April 28th, a lecture on the "Surgical Aspect of the Rontgen or Cross Rays" was delivered to the members of the Nursing Guild at Toynbee Hall by Mr. Sydney Rowlands, M.A. The lecturer performed a number of experiments, took several skiagraphs during the evening, and afterwards exhibited them by means of the oxyhydrogen lantern.—Some substantial additions have been received towards the Worcester Nurses' Home Fund; the sum total subscribed is now £1,194 16s.—A successful amateur theatrical performance was given the other day at Rushden, Northampton, in aid of the local nursing association.—The South Wimbledon District Nursing Association have intimated "to all whom it may concern," that a gift of a bicycle for the use of the nurse in her work would be extremely acceptable. We hope some kind friend will answer this request. A clergyman was lately understood to consider ladies on bicycles "emissaries of the devil." Would he change his mind if the rider were a nurse bringing comfort and help on speedy wheels?—Mr. Charles J. Darkins asks us to state that he has given up "The Sea-shell and Children's Scrap Book Missions," which he has carried on for seventeen years past.—Miss Margaret M. Fraser is the first lady to obtain the diploma of the Agricultural Science Department of the Highland Society. Miss Fraser pursued her studies at the Glasgow Technical College, and has been lecturing on butter and cheese-making for the Caithness County Council.—The Strolling Players gave a performance at St. George's Hall last week of Mr. W. S. Gilbert's drama, "Dan'l Druce," in aid of the North-Eastern Hospital for Children. The same company repeated the programme on Tuesday for the benefit of the Alexandra Hospital for Children with Hip Disease.

### Hygiene: For Nurses.

By JOHN GLAISTER, M.D., F.F.P.S.G., D.P.H.Camb., Professor of Forensic Medicine and Public Health, St. Mungo's College, Glasgow, &c.

### V.—VENTILATION—ITS PRINCIPLES—DETECTION OF AIR IMPURITIES.

We frequently speak of an empty room, but, in relation to ventilation, a room is never empty, since all its available space is filled with air. Whether its total capacity is filled with air or not will depend upon whether or not part of its air space is occupied by solid substances, such as solid articles of furniture; and since no two portions of matter can occupy the same space at the same time the solid article takes up its own bulk of air space. It is important that this simple fact should not be forgotten. Ventilation begins to have importance when a given column or body of air, bounded on four sides by walls, the openings in which bear but small proportions to the total superficies so bounded, requires to be more or less frequently changed in a given period of time. In the open, ventilation goes on unimpeded. It is only when artificial obstacles to the free interchange of currents of air are imposed that means must be devised to overcome the difficulty. It is of importance that the chimney of a room should "draw" well, else the updraught will not be sufficient to carry off the fire-smoke; hence it will cause a smoky room. How much more important, then, is the question when the processes of combustion within the body require attention. To sustain life we must breathe. An average adult male person inspires 30 5 cubic inches of air, at each breath, and he inspires about seventeen times per minute. A simple calculation will, therefore, enable us to arrive at the total amount of air used in twenty-four hours, viz., $30·5 \times 17 \times 60 \times 24 = 746,640$ cubic inches of air, or 432 cubic feet. Women and children require less than the adult male. Hence, however insignificant in percentage amount any given impurity in the air may appear, in the light of the total daily requirement, it assumes considerable importance.

In an inhabited room the chief causes of impurification of its atmosphere are (1) its human occupants, and (2) artificial lights. Both need oxygen for their sustenance. The impurities from the human being proceed from the lungs and skin, and from artificial lights, from the combustion of the lighting medium, gas or oil. As has been shown, the amount of carbonic acid gas given off from the lungs is 101 times greater than that found in the same volume of pure air, and, in addition, expired air contains organic débris from the lungs in a state of incipient putrefaction. Both are hurtful to health, the latter more so than the former, but riddance of both is at the same time desirable and necessary for health. The odour imparted to an impure atmosphere is solely due to this organic matter, and not to the carbonic acid gas, but the amount of the latter in any atmosphere is approximately a measure of that of the former.

A very simple experiment will demonstrate the need for a supply of fresh air to support combustion. Place a lighted candle on a soup plate, partly filled with water, and cover the candle with a thin glass bell-jar. The candle will burn only as long as there is air present in the jar, and will go out in a few seconds. In imagination, substitute for the candle a mouse or a man, and for the bell-jar a sealed glass case or an air-tight room, and the result will be extinction of the light of life. The Black Hole of Calcutta and the steamer "Londonderry" afford the requisite proof. Between the condition of no ventilation and one where it is free and faultless we must place our living-rooms and public buildings. Bearing in mind that the problem of ventilation consists of supplying in a given space to a given number of persons in a given time an adequate supply of fresh air, without draught, the difficulty of solution begins here. Physiological requirements indicate 3,000 cubic feet of pure air per hour as the supply to be aimed at, and the rate of movement of the supplied air must not exceed, for delicate persons, 1½ to 2 feet, and for average persons 2½ to 3 feet per second, unless the air be warmed, in which case the velocity may be raised from 5 to 8 feet per second. This is the crux of the problem, and hence it is that the cubic space per person assumes great importance. Suppose two rooms, each of 1,000 cubic feet capacity, be occupied by one and by four adults respectively, a little calculation upon the foregoing data would show that, means of ventilation being provided, the necessary amount of air could be for the one person supplied at a velocity of less than one foot (·8) per second, but that for the four persons the needed supply of air would require to have a velocity of 3⅓ feet per second; in the former case, therefore, the air would enter without draught, but in the latter it would be perceived as a draught, and would be, therefore, harmful.

The average adult gives off, in ordinary circumstances, ·6 of a cubic foot of carbonic acid gas ($CO_2$) per hour, or 14·4 cubic feet per twenty-four hours. It is an odourless gas. As has been shown, in pure air $CO_2$ amounts to ·04 per cent., or four parts per 10,000 of air. This is called original or "initial" impurity, because this percentage is found in the purest air. The disagreeable odour imparted to an imperfectly-ventilated room is due to the organic matter from the lungs of the occupants. The proportion of $CO_2$ in a room given off by respiration is called the "respiratory" impurity, and is that amount in excess of ·04 per cent. (When artificial lights are burning, as at night, they also contribute their share of this gas.) The total impurity in the air of a room is therefore made up of the $CO_2$ from the lungs of the occupants, and the $CO_2$ found in the air normally; or, in other words, the "initial" plus the "respiratory" impurity equal the total impurity. When the proportion of $CO_2$ from the lungs is less than ·02 per cent. no disagreeable odour is perceived, above that proportion odours become perceptible; thus ·04 per cent. gives a "stuffy" odour, ·06 per cent. an offensive odour, and ·08 per cent. a foul, sickening odour. Beyond this the sense of smell fails to distinguish. In the presence of the latter proportions flushings, faintness, headache, or even sickness affect the occupants. We, therefore, aim to have in our rooms not more than ·06 per cent. of this gas ($CO_2$), of which ·04 per cent. is "initial," and ·02 per cent. "respiratory" $CO_2$.

*Detection of Impurities in Air.*—The best ordinary test for these is the sense of smell, exercised on entering the room from the open air. In so far, then, as the odour of a room is in some measure an approximate test of the amount of $CO_2$ present the test is valuable. To estimate the amount by chemically accurate methods is beyond our present object. But there are two comparatively easy and approximately correct modes of doing so, called the methods of Angus Smith and of Lunge-Zeckendorff. The former consists of a series of stoppered bottles, measuring respectively 20, 10½, 8, and 6½ ounces. The chemical substance to be used is a tablespoonful (½ ounce) of clear lime-water. The test is used as follows: Into the 10½ ounce bottle put ½ ounce of lime-water, stopper, and shake contents vigorously for a minute. Should the clear liquid have become turbid, the sample of air contains more than ·06 per cent. of $CO_2$; if no turbidity results it does not contain more than this proportion. The following figures will guide to the use of the other bottles:—

| Size of bottle in ounces. | No precipitate. $CO_2$ per cent. |
|---|---|
| 20½ | ·03 |
| 10½ | ·06 |
| 8 | ·08 |
| 6½ | ·10 |

The Lunge-Zeckendorff method requires a certain chemical knowledge, but is, in practice, owing to a table of instructions which is provided by the makers with the apparatus, almost mechanical. The apparatus consists (1) of a bottle of certain cubic capacity, stoppered by an indiarubber cork perforated in two holes, through which pass two glass tubes, one of which is connected with a rubber ball of definite capacity by means of rubber-tubing; and (2) a "standard" solution of carbonate of soda, coloured pink, with an "indicator" called "phenolphthalein." To use the apparatus, a definite quantity of the prepared solution is put into the bottle, its attachments are fixed, and the rubber-ball is compressed and released alternately so as to slowly force the air through the solution in the bottle, shaking the contents after each ballful, until the pink colour practically disappears. Reference to a given table shows from the number of ballfuls used the percentage amount of $CO_2$ in the air examined. Our experience of the apparatus warrants us in saying that it is a fairly reliable approximate guide to the atmospheric $CO_2$ present. The following illustration needs no description.

Lunge Zeckendorff Apparatus for Estimation of $CO_2$ in Air.

### The Training of Male Nurses.

By Miss Darche, Lady Superintendent of the New York City Training School for Nurses, Blackwell's Island, New York.

In reply to your letter of inquiry about our male training school, I regard it simply as an expedient by which we nurse the male wards of this hospital—a considerably better expedient than the old-time method, where the old-time orderly or male nurse was in charge, but not anything like so good a method as nursing by women nurses with a paid orderly or ward attendant working under them.

As an expedient, we make the most of it, and have developed a tone and character, an *esprit de corps*, among the men which make them do the best they can. The reason they do not do better is simply because a man never can by his nature become so good a nurse as a woman by hers can. I consider nursing to be pre-eminently a woman's work, and the experience I have had in managing men's training schools and women's training schools only deepens this conviction.

I believe we get a better grade of man into our school than the man who ordinarily engages to work as ward attendant or orderly under female nurses. They are fairly industrious, some quite so; all are honest, sober, and studious. It is simply because they are men that they fall short in the housekeeping, in the close attention to detail, in the self-sacrificing spirit which is the chief characteristic of a good nurse. We prefer to take in young men from eighteen to twenty-one years of age, those who are ambitious to enter the medical profession or to study pharmacy, when, by nursing for a time at private duty after they have been trained, they have earned sufficient to put them through a student's course. Many of our men have come straight from college, some have passed the Regent's preparatory examination for the study of medicine, and quite a few are studying for that examination in addition to learning the regular lessons of the school. At the same time, we expect the men while on duty to attend strictly to ward work and nursing duties; but we give them time every day for study. They work nine hours per day as the women nurses do, thus leaving about two hours daily for recreation and study.

Our good applicants now are about equal to our vacancies. At first I had considerable difficulty in getting sufficient applicants to fill vacancies as they occurred, or as I made them occur. The men reside in bedrooms off the male wards formerly occupied by the old-time orderly, two in each room, where formerly there was one. They have a dining-room in the hospital, and a reading-room, or study, for themselves. We try to manage them in every respect the same as we do our female training school.

Were our nurses' home larger, we should undoubtedly place graduate head nurses in each ward, and nurse our male wards with our female nurses, having a ward attendant or orderly in each male ward. I should in this case try to get a good, honest, but common sort of man—one who could be called John, or Thomas, or William, as the case might be—and one who could be made to feel he was simply an all-round, useful man in the ward. I think that nothing but confusion would arise by trying to develop a male training school out of the ordinary man employed to assist women nurses in the wards; and certain confusion would arise by getting a better class of young man—one of the same social grade to work under the female nurses.

One point more. In a male training school such as ours, the best results are obtained by having it officered by women. For instance, we have no women working in the wards with the men, but the superintendent and the supervising nurse, who is a sort of general head nurse for all the wards, are both women. This has a good effect upon the general character and behaviour of the men, while the nursing and the housekeeping of the wards is the better accomplished for being done under the criticising eye of a competent woman graduate.

The Mills' Training School is the only other male training school I know of. It was organised because Mr. D. O. Mills—once sick himself, and, not being able to engage a reliable male nurse, conceived the idea of founding a training school for male nurses. He built a handsome house near Bellevue Hospital, and gave it as a nurses' home for male nurses. The male wards of Bellevue Hospital were then under the charge of the old-time orderly. Considerable reform has been effected by the change, and if the object is to educate male nurses for private duty, as in the Mills' School, and to help a certain proportion of indigent students to enter the medical profession, the male training school is undoubtedly a very good system. Sometimes reliable male nurses are required for certain cases outside in private life. Our male schools do good work in supplying this need. But, if the object is to obtain the most efficient nursing in hospitals, I think this can be best accomplished on the female training school plan, having in the male wards the ordinary man as ward worker.

### Irish Workhouse Reform.

CONFERENCE AT ST. MARTIN'S TOWN HALL.

ON April 29th a well-attended conference, under the presidency of Mr. Charles S. Roundell, was held at St. Martin's Town Hall, with the object of forming an association for the promotion of reform in Irish workhouses and infirmaries.

Dr. T. H. MOORHEAD, honorary secretary of the Irish Poor Law Medical Association, from twenty years personal experience as medical officer of a workhouse, described the existing state of affairs in these places, well named "official dustbins," in which the inmates were thrust out of sight and forgotten, to all intents and purposes dead and buried. And as long as the present general orders existed it was useless to look for real reform. These orders were framed with the intention of putting down poverty by force, and since their initiation circumstances had changed, and now the old and the helpless were the inhabitants of the workhouses. Dealing first with the nursing question, Dr. Moorhead said that he had sent circulars to the 164 Irish workhouses, and had received replies from 80 medical officers, from which it appeared that 36 infirmaries had 77 trained nurses amongst them, and 43 had no trained nurse all. In 61 of the 80 workhouses the wards were in charge of pauper inmates at night. He wished to see pauper nursing entirely done away with, and until that was done, root and branch, it was impossible to treat the sick as they should be treated. The position of the nurse too was a very serious one, because in Ireland, as in England, she had no official recognition at all, and while that was the case the better class of women would not take up workhouse nursing. Commenting upon the dietary, Dr. Moorhead stated that the staple food was bread, milk, and stirabout. As a matter of fact the medical officers were allowed by the Local Government Board to order what they wished for the patients, but it was no use to order food which there were no appliances for cooking, and which, if cooked, there were neither knives, forks, nor plates to eat it with.

Miss CATHERINE J. WOOD then gave graphic details of her personal visits to Irish workhouses, especially dwelling on the terrible condition of the aged and infirm, the hard and sometimes mouldy beds on which they lay, and their lonely locking up at night, from sunset to sunrise, with no attendance at all and no light, so that the more helpless frequently rolled out of bed and were found on the floor in the morning. The condition of the little children was even worse. The "nurseries" were entirely deficient in sanitation, and unsavoury beyond words. There was no hot or cold water laid on, and for the sick every drop of water had to be heated in a kettle.

Dr. JACOB, who represented the Irish Medical Association, insisted on the absolute necessity for a root and branch reform, and instanced some terrible examples of the scandals arising from the lack of proper care of the sick. Dr. Jacob also spoke of the system of supplying drugs as a gross swindle upon the ratepayers, the sick, and the medical officers. He did not wish to be understood as blaming boards of guardians in particular, but the effete and corrupt system they had to administer.

Speeches were afterwards made by Dr. Catherine Macguire, Lady Frances Balfour, Miss S. Crawford (Dublin), and Dr. Colles, and finally the following resolution was proposed by the Hon. Horace Plunkett, M.P.:—

That, in the opinion of this meeting, it is desirable to form an association for the purpose of promoting reform in the Irish workhouse administration; and that this association be desired to put itself in communication with the Irish Medical Association and such other bodies or committees as may be working in the cause of the reform of the workhouses.

Mr. PLUNKETT thought any political agitation was to be deprecated at the moment, but that such an association might do good by ventilating the matter and getting up a strong opinion on it. The motion was seconded by Lord MONTEAGLE, and unanimously carried.

### Novelties for Nurses.

AT MESSRS. GARROULD'S.

It is always a boon to busy people to be able to do all their shopping within one establishment. Messrs. Garrould have for some time past realised this fact, and they now offer to the nursing profession the advantage of procuring a complete outfit, as, indeed, its every accessory, at their fine emporium in Edgware Road. In these premises, which occupy a commanding position, various departments are fitted up with a view to meeting the several requirements of the nursing world. One room is for dresses and uniforms, another for cloaks, for boots and shoes, for caps and headgear, for aprons, linens, &c. But outside the actual *personnel* for nurses there is a department for instruments of every sort, chatelaines, wallets, clocks and watches, and text-books on medicine, surgery, and general nursing. All possible wants have thus been forestalled by this enterprising firm. Such is a general outline of what is to be seen here, but for more detailed particulars we will commence at the cloak department. Here each garment which Messrs. Garrould exhibit is guaranteed waterproof; they are all marked at extremely moderate prices, and there is a large selection to choose from. Private nurses, who are not hampered by circumscribed clothing, would do well to inspect some of these really charming designs. "The Rosabel," for instance, or the "Clarinda." The former of these, for summer or winter wear, is manufactured in all-wool cravanetted cashmere cloth, in blue, black, gray, or brown. It is a cloak with broad yolk back and front, full bishop sleeves, gathered at the wrist, a high upstanding collar tied with a wide bow of satin riband. Something in effect, though less exaggerated, "The Rosabel" suggests the domino form of wrap now so much in vogue. It is made in various prices according to the cloth, varying from 31s. 6d. to 37s. 6d. "The Clarinda" has a detachable cape and self-coloured velvet collar, which, when, as in one instance we noticed, it is lined with a cardinal or navy surah silk, is cheerful and bright. This is also made in fine cravanetted cashmere cloth, Melton, or winter serges. This is a still cheaper garment, obtainable for 27s. 6d. The new "Ellesmere" cloak admits of the fullest of sleeves underneath, the cape being gored and setting off somewhat from the figure. In fine all-wool serge, thoroughly shrunk, it is made in black and navy for 23s. 6d. Then dresses, like the cloaks, are shown in great variety, but nurses can have them made to order at the same rate of charges as the models, perfect fit and workmanship being guaranteed. Messrs. Garrould keep a staff of workpeople on the premises in order to execute these orders under their personal supervision, and at short notice. Of the model costumes, the new "Princess," in zephyr or washing-cotton Oxford cloth, or white French piqué, is turned out at the remarkable sum of 18s. 9d. complete! For 10s. more this same costume can be purchased in estamene or all-wool serge. The same prices also relate to "Model 19," designed with full-banded bodice and bishop sleeves. But there are other varieties of costume equally suitable for nurses' gowns, all being neat and simple. Special attention should be called to Garrould's gray all-wool "Harrisbuge" beige, of which gowns are also made here. The price for this material is, of course, a little higher, but it is well worth the difference, for the beige has many qualities which recommend it. It is manufactured in a good serviceable colour, and is both firm and light in texture. Bonnets suitable for the costumes are kept ready in stock, or are trimmed to order. The firm make a speciality of their capes, aprons, linen collars and cuffs, the materials and finish of which are excellent. Another well-stocked department contains boots and shoes for outdoor and indoor purposes, amongst the latter Garrould's celebrated "silent ward shoe."

### PATENT MILLERAIN PROOF.

IN our notice last week of the British Millerain Co.'s "Patent Millerain Proof," we omitted to state that they have a London depôt at 32, Newgate Street, E.C.

### "Truth" and the Indian Nursing Service.

By a Correspondent.

An article having appeared in *Truth* on the subject of a confidential report, I think it right, for the sake of the lady superintendent, whose name has been so roughly dragged into print, while other names have been suppressed, to give more explanation.

There is no doubt, however, as to who the aggrieved sister in question is, for from the date of her appointment being mentioned, and her having served first at Allahabad and then at Quetta, her identity is patent. Her champion, I see, speaks of "conspicuous indications of approval from her superiors." Does that mean that up till December last her conduct has always met with approval from her superiors, or that all her superiors have recognised in her the qualities of a superintendent?

A superintendent in the I.N.S. requires social qualifications and housekeeping qualifications, though she may not be selected on these grounds; but Government holds her responsible for the order and regularity of the sisters' quarters, and she has to exercise general control and supervision over the nursing sisters—her authority over them is stated to be supreme. It requires for the position a gentlewoman of tact, good temper, and firmness, so that it is most advisable that seniority should not be the only guide for promotion, and up to date there has been no precedent for it in the case of permanent appointments in the service.

Superintendents, as a rule, are selected on "grounds of experience, administrative capacity, and personal fitness," but nowhere in the regulations is there mention of being chosen on seniority alone. As the superintendent as a rule gives the tone to the nursing community it is better to go by selection; and it is also necessary to have a woman of great self control, and also a well-educated woman. We all know that the qualifications that make a good nurse don't always go to make a good superintendent; we don't need to go to India to learn that.

To prove that these appointments don't always go by seniority of service, I shall here quote a few instances, and I don't think these appointments were ever questioned by anyone.

In December, 1890, Miss Barker, without having ever served in the I.N.S. at all, was chosen by the India Office, on account of her previous career and qualifications, for a first-grade appointment in India, that of lady superintendent of the Bombay Command Indian Nursing Service.

In August, 1891, Miss James, one of the most able nurses now in the Service, was, on the same grounds, brought in as a second-grade superintendent. She now succeeds Miss Barker, at Poona, on further promotion as lady superintendent, I.N.S. Bombay Command.

In October, 1891, the following ladies now in the Service, after not quite a year's service, were made superintendents: Miss Moore, Miss Hislop, and Miss Wildman. At that time also some who were senior were passed over.

There are now some excellent senior nurses in the Service I could mention—even senior to our aggrieved *Truth* correspondent—who have not yet had the luck to hold deputy superintendents' places or acting deputy superintendents' places. As long as it is the rule to make these appointments by selection, and not seniority, our friend has no right to complain that she has been passed over. If we all got what we each thought we deserved in this world, there would be no underlings—we would all be at the top.

The fact of her being away, at the lady superintendent's visit of inspection, was her own look out. Leave, except sick leave, is entirely a voluntary thing, and I certainly think it would have been a wiser and more courteous thing to have been present at the inspection. I think it no excuse. If such an excuse were allowed, any sister who was conscious that she deserved a bad report would absent herself naturally at these times, so that the lady superintendent would be powerless to report on her. If such excuses were allowed there would soon be no discipline.

I think, for an adverse report, the lady superintendent's expressions were very mild, for all the sister admits that was reported against her was her "having a hasty temper, and also that her relations with another sister had not been pleasant" (*vide Truth*).

The practice in regard to such reports is that where a sister has had excellent reports for some years, very little notice is taken of a solitary unfavourable one. Where, however, three or four unfavourable reports have been received, those in authority are bound to take action, and such action may take the form of passing over a recommendation for promotion if the circumstances seem to warrant such a course.

It would be interesting to hear the version of the affair given by the junior sister whom she reprimanded. Why has *Truth* not taken up her cause? I don't quite understand that part. We have in the beginning of the article the complaint that she was passed over for the appointment of deputy superintendent. No nursing sister has any right to reprimand another nursing sister unless, for the time being, she is acting as superintendent, even if she was two years her senior, or three, or longer. The deputy superintendent has supreme authority over the nursing sisters, who are under her immediate supervision and control. That authority would only devolve on a nursing sister during the absence of the superintendent, owing to sickness, or temporary or privilege leave; and then it would not necessarily happen that the senior sister took her place, though, as a rule, she does in a temporary matter like that; but, if the district principal medical officer chooses, he can appoint any one of the nursing sisters to act as superintendent, she, however not being entitled to any extra salary while so acting.

The "friend" of the nursing sister concerned states that the allegation as to her having unpleasant relations with another sister is founded solely upon the fact that this sister was a junior with whom she had to find fault in the course of her duty, but does not state that she was then acting as superintendent, which would have made her case stronger. So we can only presume that, on account of her seniority in the service, she reprimanded an unfortunate junior when she had no right to; if that was the case, I am not at all surprised that the relations were "unpleasant." I should rather fancy, under these circumstances, that the matter was put very mildly.

I think myself that confidential reports—though not, perhaps, as much in this instance as in some—are matters that there is a good deal of abuse of; but in the Indian Nursing Service, as in other branches of the service, the adverse clauses in a report are always sent to the one concerned, or directions from headquarters are given to that effect. And as the officer concerned is required to put "seen" and sign them, it would be known at once if they had not been communicated to the officer. These things do happen sometimes, and the recipient is apt to consider them unjust; but the least said is soonest mended, and our aggrieved friend would have acted wisely to have either not published the matter, or, if she wanted redress, to have appealed officially against the decision of her superior; and even if the principal medical officer in the Bombay command had decided against her, she could have claimed a court of inquiry, and had the whole matter settled in Quetta and fully investigated; she would have had fair play. Miss Barker (the lady superintendent named) has left the service, and the sister is in fact mistress of the field, and I think I am right in stating that she is the only one remaining of those who served last year at Quetta at the time of the inspection.

The lady superintendent is obliged by regulations to forward her confidential report through the local office, *i.e.*, it would pass for remarks, through the hands of her immediate superior medical officer at Quetta, who could, in the same way as a commanding officer of a regiment, be able to found his opinion "from prolonged personal observation," a "daily intercourse and conversation" with the sister reported on, if he wished; then through the hands of the district and command medical officers for such remarks as these officers also choose to make or have to offer on the conduct, capabilities, and manner in which each deputy superintendent and nursing sister has performed her duties.

These three medical officers, if they had not approved of, or agreed with, the confidential report, could have offered their remarks on the back of the confidential report. Previous to this report complained of, she must have had three if not four previous confidential reports. Had all these reports recommended her for promotion I certainly do not think that the fact of her having a "hasty temper" alone, or "unpleasant relations" with one sister, would have affected the authorities in the slightest way as regarded her promotion. Selection in these matters is not made without great care and thought, and I have reason to know that when a sister does get an adverse report—which does not often occur—it is much better to say nothing about it. No one is faultless, and when once mud is stirred up by a row, it is difficult to get matters clear again as regards an individual. Of course, if the aggrieved nursing sister was acting as deputy superintendent at the time, she had every right to reprimand one under her who did wrong. Any sister can appeal if she chooses against the decision of a superintendent, but she must abide by her superior's decision until a higher authority cancels it. I quite agree with the nursing sister that she ought to have been told that the complaint had been made; but it is quite possible that the junior may have had reasons to avoid offending her senior with the "hasty temper" once more.

### Presentation.

AT the annual committee meeting of the Kent Nursing Institution, West Malling, Tunbridge Wells, and Bromley, on April 28th, the Dowager Countess of Aylesford presented Miss Ligertwood, the lady superior, on behalf of the nurses with a silver writing set, including a silver-mounted stationery case and blotting pad; and also an illuminated album, containing a letter from the nurses expressing their esteem and gratitude for her long-continued kindness and sympathy, "hoping that for many years she might hold the post she now occupies."

### Death in Our Ranks.

GREAT sorrow has been caused at the General Hospital, Birmingham, by the death, on the 29th ult., of Sister Nix. Sister Nix received her training at the General Hospital, and for the last eight years held the post of sister of a large medical ward. She had endeared herself to everyone with whom she worked, and her loss will be keenly felt. The funeral took place on the 1st inst. at the Uplands Cemetery, Smethwick. At a meeting of the Committee, held on the same day, a resolution expressing great regret at the death of Sister Nix, and sympathy and condolence with Mrs. Nix and the members of her family in their bereavement, was unanimously passed.

### Where to Go.

ST. GEORGE'S HALL, LANGHAM PLACE, W.—The Marlowe Dramatic Club are giving a special "Charity Performance" of "The Shaughraun" at this hall on Saturday, May 16th, with the very excellent object of endowing a cot in the name of the club at the Great Northern Central Hospital, Holloway. Tickets (reserved and numbered), 5s. and 3s.; unreserved, 2s. and 1s., may be obtained at the hall, or of Mr. L. H. Glenton Kerr, the secretary of the hospital.

### Metropolitan Nursing Association.

### ANNUAL MEETING.

THE Metropolitan Nursing Association held its twentieth annual meeting on Tuesday at Grosvenor House, by kind permission of the Duke of Westminster, chairman of the council. The Earl of STRAFFORD was in the chair, and there was a large attendance. Mr. Bonham Carter, Mr. Bousfield, Canon Ingram, the Rev. Dacre Craven, and Mrs. Scharlieb were on the platform, and among the audience were Lord and Lady Rothschild, the Countess of Stafford, Lady Galloway, Mrs. Dacre Craven, and Mrs. Montefiore. The adoption of the report, which was taken as read, was moved by the Earl of STRAFFORD, who commented on the fourfold objects of the association—the training and providing a body of skilled nurses to nurse the sick poor at their own homes; the maintaining a district home for nurses so trained in Central London; the supply of trained nurses to the Q.V.J.I.N. and affiliated associations; and, lastly, the raising the standard of nursing and the social position and comfort of nurses. Eighteen candidates had been admitted during the year in addition to those in the home at the beginning of 1895, the total number under training during the twelve months being 33, of whom only one proved unsuitable. The number of cases nursed was 1,468. Six Board schools had been visited daily by a nurse, and the average number of nurses resident in the home had been 12, in addition to the superintendent. The society owed its foundation and useful career mainly to the exertions of Mrs. Dacre Craven (Miss Florence Lees); it had been supported and encouraged by many charitable people, and it now rested with the public to enable it still further to increase its sphere of usefulness.

Mr. LANGTON seconded the resolution. England was, he said, in the van of all good nursing, a growth of the last fifty years, which owed its origin to Florence Nightingale. He hoped that in time the three years' standard of nursing would be universally adopted, but that must come slowly. Sooner or later the association would be able to enforce a longer training than was the case at present.

The report was unanimously agreed to.

Mr. BOUSFIELD moved a resolution pledging the meeting to support the association, and in speaking of its work, specially commented upon one development which he looked upon as a most important one, the daily visits of the nurses to certain Board schools, whereby the minor ailments of the children received proper attention, and disease and bad habits were prevented. A great improvement in the homes of the poor would result from the influence of these nurses.

Mrs. SCHARLIEB, M.D., in an excellent speech, dwelt upon the difficulties felt by poor women in times of sickness in leaving their own homes for hospital; this was where the district nurses stepped in, saved the break up of the home, and the life of the mother. Nurses were also great missionaries of light and love and goodness, and by words dropped here and there as they went about their work they exercised a most excellent influence. They brought order into homes where dirt and disorder had before reigned supreme. The Association did not attempt to cover the whole of the metropolitan area; their efforts were but as drops in an ocean—islands in a sea of insanitation—but they had a leavening effect. Mrs. Scharlieb pointed out that everybody could not do the nursing work. There were very few people like Florence Nightingale and Mrs. Dacre Craven, but others could give the money, without which the work could not be done, and she urged the duty of giving tithes, a hundred pounds in every thousand, to charity. She was not preaching a gospel of asceticism, but between absolute self-renunciation and easy self-indulgence there was a wide difference. People's own wants increased with their incomes, but their charitable offerings not at all in the same ratio.

Speeches followed from Canon Ingram, Mr. Bonham Carter, and others. After the meeting there was an adjournment to the Home, 23, Bloomsbury Square, for tea and coffee.

### Everybody's Opinion.

[Correspondence on all subjects is invited, but we cannot in any way be responsible for the opinions expressed by our correspondents. No communications can be entertained if the name and address of the correspondent is not given, or unless one side of the paper only be written on.]

### PRIVATE CONVALESCENT HOMES.

Miss Hall Hall writes: May I be allowed, through the medium of your columns, to get at some knowledge regarding the existing state of supply and demand in the matter of small private convalescent homes? I have had a small private home for some years intended to meet the needs of rather better class people, such as those engaged in business, tuition, or nursing, and especially such as are needing care and rest after operation or treatment in a hospital, and are able and willing to pay a moderate sum for themselves, or are paid for by some lady personally interested in them, but who would not care to apply for more public charity. With this view I have taken a pretty cottage with six available beds in a very healthy and picturesque locality, but up to the present I have only had an average of three per week throughout the year, and the payment at from 10s. 6d. to 15s. per week only meets about two-thirds of the cost. For nine months of the year two beds are almost always occupied by convalescents from a women's hospital, paid for by one lady. My object in writing is to get information on the following points. Are there more of such homes than are needed? If not, how is the class to be reached for whom the home is intended? I should be grateful for any reliable information on the subject. [We can only suggest advertising. Undoubtedly there does exist a very large class of people who would be glad to avail themselves of such an opportunity for rest and change at a small charge.—Ed. *T.H.*]

### Appointments.

Infectious Diseases Hospital, Wallsend.—Miss Nellie Smith has been appointed nurse-matron at this hospital. Miss Smith was trained at the Paisley Infirmary, and has since been on the staff of the Sunderland Nursing Institute for over five years. She has our best wishes for success in her new work.

Gorleston Cottage Hospital.—Miss Isabel Frith has been appointed to the post of Nurse-Matron at this hospital. She was trained at Addenbrooke's Hospital, Cambridge, and then worked as night nurse at the Manchester Children's Hospital for two years. Afterwards Miss Frith was for four years matron at Lytham Cottage Hospital. We wish her all success.

Camberwell Infirmary.—Miss S. E. Kidson has been appointed Assistant Matron and Superintendent of Nurses at the Camberwell Infirmary. Miss Kidson was trained at St. Mary's Hospital, Paddington, and has since been employed at the St. Saviour's Union Infirmary, East Dulwich Grove, first as sister and for the last two years as night superintendent.

### Minor Appointments.

Metropolitan Hospital, Kingsland Road.—Miss Julia Moule has been appointed out-patient Sister at this hospital. She received her training at Charing Cross Hospital and at the Metropolitan Hospital, where for some time she has worked as out-patient nurse. We congratulate Miss Moule on her promotion.

Children's Hospital, Luton.—Miss Florence M. Johnson has been appointed Head Nurse at the above hospital. She was trained at the Royal Albert Edward Infirmary, Wigan, and has, amongst other appointments, held the post of sister at the British Hospital, Port Said.

Royal Northern Sea Bathing Infirmary, Scarborough.—Miss Kate Heaton and Miss Florence Allcroft have respectively been appointed Charge Nurses of the male and female wards of this institution.

### Wants and Workers.

Nurse J., 42, Elgin Avenue, Maida Vale, would be very glad to receive any left-off clothing for a poor girl just going out to service.

"A Future Jubilee Nurse" asks: Has any reader of The Hospital any or all of the following books to dispose of second-hand, or can they say where they may be procured? Collie on "Fevers," Church on "Food," Willoughby on "Hygiene," and Mitchell Bruce on "Materia Medica."

### Notes and Queries.

The contents of the Editor's Letter-box have now reached such unwieldy proportions that it has become necessary to establish a hard and fast rule regarding Answers to Correspondents. In future, all questions requiring replies will continue to be answered in this column without any fee. If an answer is required by letter, a fee of half-a-crown must be enclosed with the note containing the enquiry. We are always pleased to help our numerous correspondents to the fullest extent, and we can trust them to sympathise in the overwhelming amount of writing which makes the new rules a necessity. Every communication must be accompanied by the writer's name and address, otherwise it will receive no attention.

### Queries.

(28) *Unreturned Testimonials.*—Some three months ago I applied for a position as Nurse-Matron, in answer to an advertisement in The Hospital. I have received no notice of the appointment having been made, nor has the secretary taken any notice of a letter asking for the return of my photo and testimonials. Can you advise me?—*M. E. L.*

(29) *Training.*—Information wanted about the training of probationers.—*M. A.*

(30) *Army Nursing.*—How would it be possible for me to get an appointment on active service as nurse? I wish very much to go out to Egypt.—*Uncertificated* and *Nurse Helen.*

(31) *Training.*—I am anxious to train as a nurse, and should be glad of advice as to the right steps to take. I am just twenty-two.—*Lonely.*

(32) *Monthly Nursing.*—Will you tell me where I can obtain training as a monthly nurse without paying a premium, and what is the usual length of time required?—*G. M.*

(33) *Leprosy.*—I am wishing to study leprosy and work amongst the lepers at Robben Island or some other colony under English supervision. I have gone through the medical curriculum. To whom should I apply?—*Inquirer.*

(34) *Book-keeping.*—Can you tell me if there are any such posts as book-keepers in hospitals and institutions, and how they can be obtained?—*Secretary.*

(35) *Medical Dictionary.*—Please tell me the best illustrated pronouncing medical and surgical dictionary, with price and publisher?—*Nurse Surrey.*

(36) *Nursing of Infants.*—Can you tell of any place where children's nurses can be thoroughly taught to manage infants from the birth?—*Nurse L.*

(37) *General Training.*—Where can I obtain two years' good general hospital training?—*N. F.*

*Patents.*—Will Nurse Mona send her name and address? See note at the head of this column.

### Answers.

(28) *Unreturned Testimonials* (*M. E. L.*).—We should suggest your writing again to the secretary, and if you receive no reply write through a solicitor. We constantly warn nurses that original testimonials should never be sent in with an application. In the first place it is running the risk of losing them, and, in the second, it is expecting rather too much of the hospital authorities to return numberless testimonials. It is not always customary to inform unsuccessful candidates that an appointment has been filled.

(29) *Training* (*M. A.*).—You will find all the information you require, with the regulations for the admittance of probationers into various hospitals, in "How to Become a Nurse" (Scientific Press, 428, Strand, W.C.).

(30) *Army Nursing* (*Uncertificated* and *Nurse Helen*).—Nurses who have trained for three years in a good general hospital are eligible for appointment on the army or navy nursing services, and should apply to the Director-General of the Army Medical Department, Victoria Street, Westminster, S.W. It is only in this way that foreign appointments can be obtained, and naturally it is not the new comers who are sent to "the seat of war." The nurses who are sent on foreign service are chosen from among the most experienced sisters, those who are well accustomed to army nursing in times of peace.

(31) *Training* (*Lonely*).—You are young to enter a general hospital, where probationers are seldom admitted under twenty-five, though there are exceptions. You had better write to the matrons of the children's hospitals, and ask for particulars and a form of application. By way of reference you only need to give the name of someone who knows you—your doctor or clergyman, for instance. "Testimonials" are not needed.

(32) *Monthly Nursing* (*G. M.*).—Fees are required at all lying-in hospitals for the training given. The time varies at the different institutions from one month to three, but the latter is the shortest time that ought to be given to learning so important a branch of nursing, if the knowledge gained is to be thorough. If you write to the manager (Scientific Press, 428, Strand, W.C.), he will recommend a book containing all particulars.

(33) *Leprosy* (*Inquirer*).—We are sorry your query could not be inserted last week through want of space. You could probably obtain the information you require on application to Dr. P. S. Abraham, 2, Henrietta Street, Cavendish Square.

(34) *Book-keeping* (*Secretary*).—Certainly clerks are employed in the secretary's and steward's offices in hospitals and institutions, for book-keeping and secretarial work generally, and vacancies would be advertised in the usual way. We hope you have received the copy of The Hospital to which you refer.

(35) *Medical Dictionary* (*Nurse Surrey*).—"Quain's Dictionary of Medicine" (illustrated), £2 net, Longmans; or a complete Pronouncing Medical Dictionary, by J. Thomas, M.D., price 10s. 6d., Lippincott.

(36) *Nursing of Infants* (*Nurse L.*).—Write to the Norland Institute, 29, Holland Park Avenue.

(37) *General Training* (*N. F.*).—You will find a complete list of hospitals in Burdett's "Hospitals and Charities" (Scientific Press, 428, Strand. Write direct to the matrons for particulars.

## Figures and Tables

**Figure f1:**
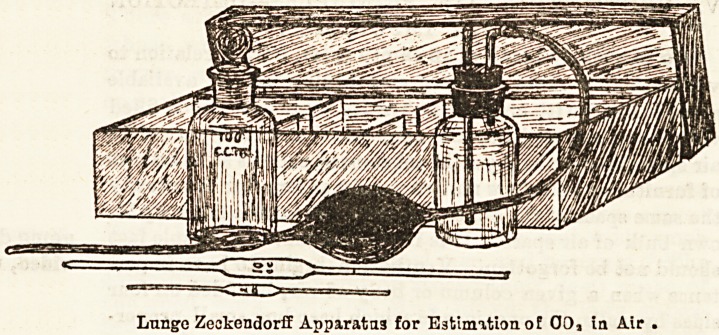
Lunge Zeckendorff Apparatus for Estimation of CO_2_ in Air.

